# Cost–benefit analysis of kidney transplant in patients with chronic kidney disease: a case study in Iran

**DOI:** 10.1186/s12962-022-00372-1

**Published:** 2022-07-29

**Authors:** Farzaneh Abdi, Cyrus Alinia, Ali Taghizadeh Afshari, Hasan Yusefzadeh

**Affiliations:** 1 Department of Health Economics and Management, School of Public Health, Urmia University of Medical Sciences, Urmia, Iran; 2 Nephrology and Kidney Transplant Research Center, Clinical Research Institute, Urmia University of Medical Sciences, Urmia, Iran

**Keywords:** Economic evaluation, Cost benefit analysis, Chronic kidney disease, Willingness to pay, Kidney transplant

## Abstract

**Background:**

Chronic kidney disease (CKD) is a health problem due to its increasing prevalence and imposes a significant economic burden on the health system. This study aimed to analyze the cost–benefit of kidney transplantation through the valuation of patients with ESRD for a kidney transplant and its costs to help decide this regard.

**Material and methods:**

This study was a descriptive-analytical and cross-sectional economic evaluation study of health interventions performed in Imam Khomeini Hospital in Urmia from the patient’s perspective. The records of kidney recipients were used to calculate the direct costs of kidney transplantation based on the government tariff rate in 2021. The willingness to pay for kidney transplantation (benefit) was measured through a questionnaire and with a contingent valuation method from 266 samples of patients with ESRD. The questionnaire designed by the researchers had four scenarios with different chances for kidney transplant success. Validation and test–retest methods were used to check the validity and reliability of the questionnaire. Stata software was used to estimate the regression of the factors affecting the willingness to pay and the kidney transplant demand function.

**Results:**

The average cost of a kidney transplant was $877.4. The average willingness to pay for a kidney transplant for four scenarios was estimated at $4733. The mean cost–benefit ratio (BCR) and net present value (NPV) for the four kidney transplant scenarios were 5.39 and $3855. The variables of employment status, awareness of kidney function, number of years with ESRD, insurance coverage, and patients’ income significantly affected their willingness to pay. However, the effect of other variables was not significant. The absolute value of price elasticity of kidney transplant demand was also equal to 2.13.

**Conclusion:**

According to the cost–benefit analysis indexes, the study results showed that a kidney transplant has a net positive benefit for all levels of its probability of success, so the willingness to pay or valuation of patients is about five times the cost of a kidney transplant. Also, the demand for kidney transplantation shows the high sensitivity of the demand for this service to the price. Therefore, preparations for kidney transplantation in patients with ESRD should be considered in situations where the price and cost of transplantation change. The results can help health policy-makers decide to allocate financial resources more efficiently.

**Supplementary Information:**

The online version contains supplementary material available at 10.1186/s12962-022-00372-1.

## Introduction

Today, despite the increase in life expectancy, chronic diseases are one of the significant health issues in the world. People with these diseases have to change their role from ordinary people in everyday life to a person with permanent patient roles. They are always under the supervision of treatment groups. Chronic kidney disease (CKD) is one of the chronic diseases that causes a person to constantly play the role of a patient in life due to his health condition and disease and its treatment [1].

CKD is a stage in which kidney function reaches less than 50% of its standard capacity [2]. If the kidneys cannot function more than 10–15% of their standard capacity as the end-stage renal disease (ESRD) is considered. End-stage renal disease can cause complications, including anemia (not enough red blood cells to carry oxygen throughout the body), bone disease, brain damage, edema (swelling), fluid in and around the lungs, high levels of certain minerals (potassium or phosphorus), infections, nerve damage, seizures, stroke. At this stage, kidney transplant or dialysis of the type of hemodialysis or peritoneal dialysis becomes necessary for the survival of the person [3].

In general, the incidence of this disease in most countries is more than 200 cases per 1 million people in a year. Due to its increasing prevalence and high economic burden, it has become a problem and a threat to global health [4, 5].

Today, one of the main goals of governments is to organize, provide and finance health services for all members of society [6]. Lack of adequate funding is currently one of the most common problems in the health sector, so discussing how to finance health services is one of the main challenges for governments and providers [7]. In this regard, the exceptionally high costs of treating ESRD and eliminating subsidies for this disease can reduce government spending in the short term. However, in the long run, if the disease is not controlled, the direct and indirect costs to patients (costs of absenteeism, reduced productivity, and production) will impose a significant financial burden on patients, their families, and society. Currently, basic and complementary health insurances largely cover kidney transplant costs in the Iranian health system. In addition, the gift of self-sacrifice is paid to kidney donors, part of which is paid by the recipient's family and part by the Ministry of Health and the Foundation for Special Diseases. This amount of gift is now 1160$.

The health and economic consequences of medical interventions and the costs and financing of these interventions are considered by health managers and economists, examined in economic evaluation studies such as cost-effectiveness, cost-utility, and cost–benefit. Cost–benefit analysis is one of the economic evaluation methods in which both costs and consequences of interventions are measured on a monetary scale. In the CBA approach, if the benefits of an intervention or program outweigh the costs, that intervention or program will be preferable to others and therefore increase social welfare [8].

Cost–benefit analysis is especially useful for valuing goods and services that show health and non-health consequences [9]. Because a successful kidney transplant prolongs the patient’s life and increases productivity and income, it is a clear example of the above goods. Therefore, valuing the consequences of kidney transplantation using monetary units and its cost–benefit analysis seemed to be a practical solution.

The increase in new dialysis cases and demand for services by this group of patients and the dramatic increase in the price of medical services make it necessary to choose the most cost–benefit of treatment method. The cost–benefit study can also help rank alternative treatments for diseases according to the different benefits and efficiencies of treatment methods and their specific cost level. Accordingly, the basis for measuring the benefits of alternative treatments must be determined [10]. The benefits or consequences of a kidney transplant can be measured by estimating the willingness to pay for each of the designed scenarios based on the likelihood of successful treatment. In general, preferences and willingness to pay are common ways to measure the value of goods or outcomes that reflect the total utility of health and non-health outcomes [11]. In other words, in the willingness to pay method, the amount of willingness to pay by individuals is calculated to get rid of disease or health problems.

Economists often use the Contingent Valuation Method (CVM) to derive their willingness to pay. In the CVM, individuals are asked to set a price for a commodity that may not be purchased (non-consumable). In this method, the individual is asked how much he or she is willing to pay to receive the hypothetical service in question [12]. The studies of Farabi et al. [19], Darvish et al. [6], Killing (2017), and Herold [23] also used the willingness to pay approach to analyze the cost–benefit of the service.

According to the Ministry of Health’s Center for Specific Disease Management and Kidney Transplant statistics, about 100,000 people in the country with advanced CKD are being treated with blood and peritoneal dialysis. The pain of dialysis and its financial burden for patients with ESRD should be noted in those on dialysis. According to statistics, the cost of each dialysis session for a patient is about $29 and equivalent to $4176 per year [13]. By calculating the population of thousands of dialysis patients in the country, it is possible to predict the heavy burden of this disease on the country’s health economy. In Iran, unlike other developed countries, financial support is much less and requires increasing attention from insurance companies and the government. Therefore, an analysis of the economic aspects of ESRD seemed necessary due to its prevalence and high treatment costs in Iran.

This study was conducted in Urmia, the center of kidney transplantation in the northwest of the country, with the aim of cost–benefit analysis of receiving it by assessing the valuing of patients with ESRD for a kidney transplant and hospital costs of transplant. Finally, a successful probability of kidney transplantation with more positive net benefits was identified for subsidized resources and investment allocation.

## Methods

This study was an economic evaluation study of health interventions conducted in Urmia. Urmia is one of the centers of kidney transplantation in the country, so this city was selected for the study. To accurately estimate the direct costs associated with a kidney transplant, including the cost of visits, surgery, tests, ultrasound, and pharmaceutical items were used from the records of kidney recipients, kidney transplant tariffs in 2021, and comments of kidney surgeons.

The maximum willingness to pay patients under four specific scenarios, designed based on the probability of kidney transplant success, was measured through a questionnaire using CVM and bidding price acquisition as double-bounded dichotomous-choice through face-to-face interviews with 266 patients with CKD (Additional file 1). This researcher-made questionnaire included information about kidney transplantation, the socioeconomic status of participants, and the four scenarios of a kidney transplant to assess the willingness to pay in each of these scenarios. The differences in the studied scenarios were 10, 30, 70, and 99% chance of a successful transplant. These probabilities were designed based on transplant specialists’ opinions to measure true willingness to pay if they know the chances of transplant success. In the double bounded method, each respondent is offered two amounts; the second amount depends on the answer to the first offer. If the answer to the first amount is positive, the second amount that is higher than the first amount will be provided, and if the answer to the first offer is negative, the amount of the second offer that is less than the amount of the first offer will be presented. Batman states that the second bid (in case of a positive response to the first bid) should be twice the amount of the first bid, and the amount of the second bid (if the answer to the first bid is negative) should be half the amount of the first bid [14]. For example, in the first scenario, the patient with ESRD is told that if the cost of kidney transplantation is 1549$ and the success rate of the transplant is 10%, how much would you like to pay? People who answer no to the first offer will be offered a smaller amount, and patients who answer yes will be offered a higher amount.

Multivariate regression was used to measure the factors affecting the willingness to pay. The dependent variable is the willingness to pay (WTP). The independent variables (X) include awareness of kidney function, stage of CKD, and socioeconomic variables such as household income, age and sex, marital status, education, basic and complementary health insurance status, and employment status. ${\text{X}}_{\text{i}}$ are the model’s explanatory variables, β is the coefficient vector, and ${\upvarepsilon }_{\text{i}}$ is the error term. ${\text{WTP}}_{\text{i}}$ is also willingness to pay of the ith person.

$${\text{WTP}}_{\text{i}}={{\upbeta X}}_{\text{i}}+{\upvarepsilon }_{\text{i}}.$$

The demand amounts for kidney transplantation were calculated at different price levels to extract this demand function. The following equation estimated the linear demand function using Ordinary Least Squares (OLS).

All data were converted to a logarithmic form to better estimate the demand function, in which case β represents the elasticity.

$${{\text{Q}}_{\text{i}}} = \alpha - \beta \;\ln {{\text{P}}_{\text{i}}} + \upvarepsilon,$$

where Q is the number of people accepting the proposed price, P is the proposed and accepted price, β is the slope of the demand function, α is the intercept of the demand function, and ε is the statistical disturbance component.

Pagan test was used to analyze the data for the presence of variance’s heteroscedasticity [15] via Stata software version 14.

Finally, to help policy-makers and managers decide on the optimal and correct allocation of limited resources of the health system, the cost–benefit analysis of kidney transplantation was performed using the benefit–cost ratio (BCR). In this study, to calculate the above ratio, the present value method was used, and the present value of the benefits of the studied scenarios was divided by the present value of kidney transplant costs [16].

$${\text{B}}/{\text{C}} = {\text{PV}}\left( {{\text{Benefits}}} \right)/{\text{PV}}\left( {{\text{Costs}}} \right) = {\text{PV}}_{\text{B}}/{\text{PV}}_{\text{C}}.$$

Generally, If B/C ≥ 1 or B-C ≥ 0 is an economic intervention and resource allocation is a higher priority for that intervention, and if B/C < 1 or B-C < 0 is not an economic intervention.

## Results

### Descriptive statistics

According to Table 1, among the 266 patients studied, 153 (57.52%) were male, and 113 (42.48%) were female. Out of the total sample, 157 patients (59.02%) were unemployed, and 106 participants (40%) were employed. More than 39% of the study samples, i.e., 106 patients, had rural health insurance coverage, followed by social security insurance, with more than 92 patients (34%) was in the next rank. Out of the four age groups of patients classified in this study, the highest statistics belonged to the age group of 45–64 years with more than 132 patients (49%), and the lowest belonged to the age group of 65–75 years which was less than 1% of total samples that were equivalent to 1 patient. More than 95% of the patients participating in the present study stated that their most important source of preparing for kidney transplantation is buying from a healthy person. The priority of about 3% of the study samples was a gift from the deceased. 33.83% of the study samples, equivalent to 90 patients, stated that their kidney functions were less than 15%, and 1.5% of the study population (4 patients) were unaware of their kidney function. The average income of patients participating in the study is $482. Also, the minimum and maximum incomes of the studied sample were $58 and $1740, respectively.

**Table 1:** Patients’ information to estimate willingness to pay

Variables	Number	Frequency (%)
Gender		
Male	153	57.52
Female	113	42.48
Employment status		
Unemployed	157	59.02
Employed	109	40.98
Health insurance status		
No insurance coverage	4	1.5
Health insurance (rural insurance)	58	21.58
Health insurance (other than rural insurance)	106	39.85
Social security	92	34.59
Armed forces	4	1.5
Other	2	0.75
Age		
0–19 years	14	5.26
20–44 years	119	44.74
45–64 years	132	49.62
65–75 years	1	0.38
Preference for a kidney preparation		
Buy from a healthy person	255	95.86
Gifts from the deceased	8	3.01
Gifts from family and friends	3	1.13
Awareness of kidney function		
I don’t know	4	1.5
Less than 60%	28	10.53
Between 30 and 50%	65	24.44
Between 15 and 30%	79	29.7
Less than 15%	90	33.83
Average income ($)	266	482 ± 370

### Kidney transplant cost

The mean direct cost of a kidney transplant was $877 in 2021. Indirect costs include travel and accommodation costs, and absenteeism from work was not considered.

### Willingness to pay for a kidney transplant

Table 2 shows the willingness to pay of chronic kidney patients by scenarios. The difference between these scenarios was a 10, 30, 75, and %99 chance of success of kidney transplantation. According to the scenarios and chance of success, the average willingness to pay was 3650, 4130, 5387, and $5764. The average willingness to pay for a kidney transplant for four scenarios was $4733. Also, the lowest and highest willingness to pay for a kidney transplant were 725 and $9859, respectively.

**Table 2:** Cost–benefit analysis of kidney transplant in patients with CKD in four scenarios

Subject	Amount ($)				Average total of four scenarios
	Scenario1	Scenario 2	Scenario 3	Scenario 4	
Average willingness to pay	3650	4130	5387	5764	4733
Average kidney transplant costs	877	877	877	877	877
Benefit cost ratio (BCR)	4.16	4.71	6.14	6.57	5.39
Net present value (NPV)	2773	3253	4510	4887	3856

### Cost–benefit analysis of kidney transplant

The values of net present value (NPV) and cost–benefit ratio (BCR) used in this study to analyze the cost–benefit Analysis of kidney transplantation were calculated according to the scenarios in the table below. The average BCR for kidney transplantation was obtained from the average BCR of all Patients for the four scenarios equal to 5.39. In addition, the average NPV for the four kidney transplant scenarios was estimated at $3855.

### Kidney transplant request function

Table 3 shows the details of the estimated kidney transplant demand function, which is derived from the willingness to pay of patients with renal failure for receiving a transplant. The price coefficient (β) shows the slope of the demand function as well as the price elasticity of kidney transplant demand which is equal to − 2.13. The R^2^ index, which is one of the model’s fitting indices and shows the dependent variable’s predictive power based on independent variables, is 0.64 for this model. The value of this index is between zero and one, and if it is more than 0.6, it shows that the independent variables have been able to explain the changes of the dependent variable to a large extent. Therefore, the selected model for estimating the kidney transplant demand function has a good fit. F-statistic also indicates the overall significance of a regression. Breusch-Pagan’s null hypothesis based on a constant variance in this model was not rejected, and the data did not have the problem of variance heteroscedasticity.

**Table 3:** Results of estimating the kidney transplant demand function

Variable	Coefficient (β)	Standard error (SE)	t-statistic	P-value
p	− 2.128864	0.5371697	− 3.96	0.003
Intercept	12.89876	2.340703	5.51	0.000
F	15.71			
P	0.0033			
R^2^	0.6357			
Adjusted R^2^	0.5952			

**Fig. 1:**
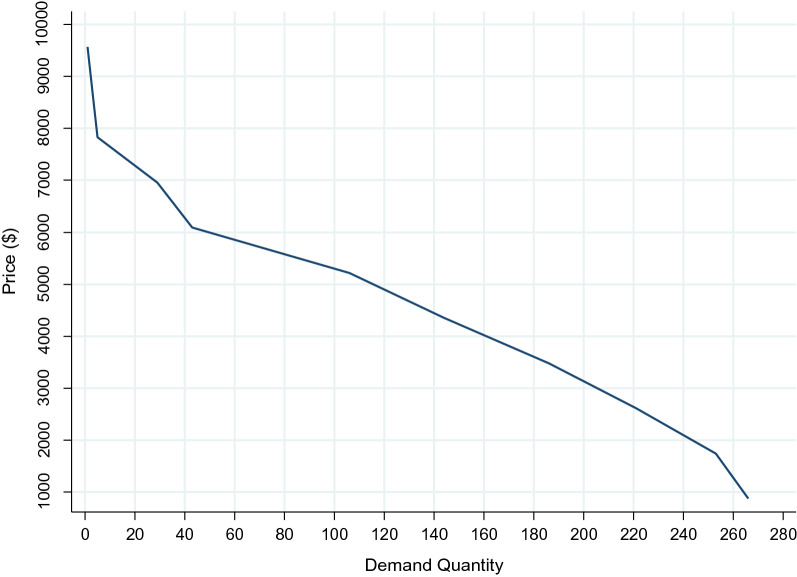
Kidney transplant demand function curve

As shown in Fig. 1, the demand function curve of kidney transplantation has a negative slope. In this curve, the vertical axis shows the proposed price, and the horizontal axis shows the amount of demand, which is the number of patients accepting the proposed price for a kidney transplant.

### Factors affecting the willingness to pay for a kidney transplant

The results of the multivariate regression model of factors affecting patients’ willingness to pay for a kidney transplant service are presented in Table 4. In this regression, the dependent variable was the willingness to pay and the independent variables were age, sex, employment, years with kidney failure, education level, awareness of kidney function, basic health and supplementary insurance status, doctor’s influence on kidney transplant, preference for a kidney preparation, and monthly income.

**Table 4:** Results of linear regression estimation of factors affecting patients’ willingness to pay for a kidney transplant

Variable	Coefficient (CI 95%)	p-value
Sex		
Male	Ref.	
Female	0.026 (− 0.023, 0.0758)	0.296
Age group		
0–19	Ref.	
20–44	0.188 (0.72, 0.30)	0.002
45–64	0.1405	0.015
65–75	0.215	0.281
Years with kidney failure		
Two years or less	Ref.	
Five years or less	0.175 (0.10, 0.25)	0.000
Over 5 years	0.2635	0.000
Education level		
Elementary and secondary education	Ref.	
High school diploma	− 0.055 (− 0.12, 0.11)	0.102
Academic degree	− 0.0165	0.586
Awareness of kidney function		
Don’t know	Ref.	
Less than 60%	− 0.12 (− 0.34, 0.089)	0.249
Between 30 and 50%	0.892	0.412
Between 15 and 30%	0.3126	0.005
Less than 15%	0.4041	0.000
Supplementary insurance status		
No	Ref.	
Yes	− 0.062 (− 0.138, 0.013)	0.109
Doctor’s influence on kidney transplant		
Don’t know	Ref.	
Mostly yes	− 0.27 (− 0.61, 0.65)	0.412
Definitely yes	0.1348	0.239
Basic health status		
No insurance coverage	Ref.	
Health insurance (rural insurance)	0.426 (0.19, 0.65)	0.000
Health insurance (other than rural insurance)	0.3944	0.001
Social security	0.405	0.001
Armed forces	0.3034	0.04
Other	0.3384	0.064
Monthly income (log)	0.2727 (0.2158, 0.3297)	0.000
Preference for a kidney preparation		
Buy from a healthy person	Ref.	
Gifts from the deceased	0.060 (− 0.20, 0.08)	0.412
Gifts from family and friends	0.1402	0.239
Employment status		
Unemployed	Ref.	
Employed	0.101 (0.04, 0.15)	0.000
Constant	14.37791 (13.43, 15.33)	0.000
Number of observations	266	
R^2^	0.8635	
Adjusted R^2^	0.8499	
F	63.51	
Prob	0.000	

More than 86% of the dependent variable changes are the willingness to pay of patients for kidney transplantation by independent variables included in the model (R^2^ = 0.8635). F-statistic also shows the overall significance of regression (F = 63.51).

## Discussion

Based on the study results, the cost of a kidney transplant was estimated at $877, and the average willingness to pay for the four designed scenarios was $4733. The average net present value (NPV) and cost–benefit ratio (BCR) are $3855 and 5.39. The values obtained for the net present value are positive for all four scenarios, and their number is high. Also, the values of the cost–benefit ratio of kidney transplantation for the four scenarios are greater than one; it can be said that all four scenarios have a high cost–benefit. The cost of a kidney transplant is far less than the value of its benefits in all four scenarios.

The high values of the NPV and BCR relations might be that patients with ESRD can get rid of the pain and complications of chronic dialysis and, consequently, its direct and indirect costs once by spending on transplant costs. In addition, as proven in previous studies, kidney transplant improves the quality of life and life expectancy of such patients [9, 13]. The results of the Axelrod study also showed the cost-effectiveness of all kidney transplant options, i.e., transplantation from a living and deceased donor, compared to dialysis due to improved survival [17], which confirms the results of the present study.

The study’s findings indicated that the patients’ willingness to pay was affected by their chances of a successful kidney transplant; as the probability of its success increases, the willingness to pay of patients also increases. In the first hypothetical scenario, where the probability of success was %10, the average patients’ willingness to pay was estimated at $3650. According to the average cost of a kidney transplant, which is equal to $877, it is clear that in this scenario, kidney transplantation, despite its low success rate, is cost–benefit. A BCR ratio greater than one (4.16) and a positive NPV ($2772) confirms this.

The average willingness to pay in the second and third scenarios was obtained at 4130 and $5387. In these scenarios, the values of BCR and NPV relations, which are [4.71 and 6.14] and [3253 and $4509], respectively, show that kidney transplant is also cost–benefit. The results of a kidney transplant's third and fourth scenarios have a higher net positive benefit than the first scenario. In the fourth scenario, the willingness to pay was $5764, and the values of NPV and BCR were equal to $4886 and 6.57. This subject shows that a 99% chance of success for a kidney transplant is worth about 6.5 times the actual cost of a transplant for patients. It makes the patients more valuable if they are sure of the result of the transplant.

In different studies such as Palumbo and Darvishi et al., the patients’ willingness to pay for the desired service has increased with increasing the chance of successful treatment and is consistent with the results of this study [6, 18]. Therefore, the probability of transplant success can be one of the most critical factors affecting the patients’ willingness to pay for this service.

Various factors influence the patients’ willingness to pay for kidney transplants. In this regard, Patients’ income has a positive and significant effect on their willingness to pay. However, the income elasticity (income coefficient in the estimated model) is less than one; therefore, with a one percent increase in patients’ incomes, their willingness to pay increases by less than one percent. The result is consistent with dissimilar studies of Farabi and Darvishi [6, 19]. Because the amount of income elasticity is greater than zero and less than one, it can be said that a kidney transplant is a necessary service for patients with ESRD.

In this study, respondents and surveyed patients, knowing that the older patients were, the lower the chance of successful transplant, were less likely to pay even in similar scenarios than younger patients and the reference group. This result is consistent with other existing but dissimilar studies by Darvishi, Farabi, and Mayer [6, 19, 20].

Awareness of kidney function and the number of years with ESRD positively and significantly affected the patient's willingness to pay. This result was consistent with the results of a study by Jensen et al. and Tan et al., which found that kidney transplant was better in the treatment of patients with end-stage renal disease than dialysis [21, 22]. Having insurance coverage has a positive and significant effect on the patients’ willingness to pay with ESRD. People with insurance coverage expect insurance to contribute to kidney transplant costs compared to those without health insurance, so they tend to pay more than the first group, the uninsured. The result of Farabi’s study in this regard was similar to the result of the present study [19].

The employment status variable had a positive and significant effect on an individual’s decision about the willingness to pay in this study, and the result was consistent with Herold’s study [23].

Other variables of supplementary insurance status, Preference for a kidney preparation, doctor’s influence on kidney transplant, education level, and gender of patients with ESRD were not statistically significant. The results obtained for these factors in the present study did not confirm the results of other previous studies.

Studies have evaluated the benefits of kidney transplantation in conditions where the benefit has been measured with a willingness to pay approach have not been performed so far. Therefore, some comparison with previous studies was somewhat impossible.

In this study, the absolute value of price elasticity of 2.13 for kidney transplant demand indicates that the demand for this service was elastic and sensitive to price changes; if the price increases by one percent, demand will decrease by more than one percent. Given that dialysis is a substitute for a kidney transplant in patients with ESRD; therefore, patients are more sensitive to the price and cost of a kidney transplant.

The exclusion of indirect costs, such as travel expenses and lost productivity of patients and their companions in calculating kidney transplant costs, was one of the limitations of this study.

In this study, due to the relationship between insurance coverage and the willingness to pay for a kidney transplant, basic insurance organizations can be considered one of the sources of kidney transplant financing. In particular, the benefits of receiving this service go directly to those organizations by reducing future dialysis costs.

Given the relationship between willingness to pay and patients’ monthly income to receive a kidney transplant, low-income people have a low willingness to receive a transplant due to financial problems. However, these people are far more vulnerable to high-income people with decreased kidney function. Therefore, it is suggested that low-income people be provided with support measures to receive this service.

## Conclusion

Based on the results of this study, kidney transplant had a net positive benefit and was economically justified in all scenarios; on average, patients valued a kidney transplant at about five times the cost. The high economic justification of kidney transplantation could be that receiving this service by patients with ESRD and improving their quality of life is expected to achieve significant savings in financial resources by reducing the need for future medical expenses of this disease. Also, in this study, the variables of employment status, awareness of kidney function, number of years with ESRD, health insurance status, and patients’ income significantly affected their willingness to pay, and the effect of other variables was not significant. Estimation of the kidney transplant demand function also showed that patients' demand was highly elastic and sensitive to price changes. The results can help health policymakers’ decisions on the optimal allocation of financial resources (including public resources, insurers’ resources, and household budgets) to the provision of the service under study.

## Supplementary Information

**Additional file 1.** Translated questionnaire.

## Data Availability

The essential data is available in the article and we can provide upon request.

## Abbreviations

CKD: Chronic kidney disease
BCR: Cost–benefit ratio
WTP: Willingness to pay
ESRD: End-stage renal disease
NPV: Net present value
