# Supplementary material for: Cost–benefit analysis of kidney transplant in patients with chronic kidney disease: a case study in Iran
Source: Cost Eff Resour Alloc. 2022 Jul 29;20:37. doi: 10.1186/s12962-022-00372-1 (PMC9338670; doi:10.1186/s12962-022-00372-1)
Supplement: Supplementary file 1 — Additional file 1. Translated questionnaire. [file 12962_2022_372_MOESM1_ESM.docx]

**Translated** **questionnaire**

The following questionnaire is used to conduct a study entitled Cost-benefit analysis of kidney transplantation in patients with chronic kidney disease based on the willingness to pay approach.

**Section 1: Background information**

Definition of Chronic Kidney Disease

CKD is a stage in which kidney function reaches less than 50% of its standard capacity. If the kidneys cannot function more than 10-15% of their standard capacity as the End-Stage Renal Disease (ESRD) is considered. End-stage renal disease can cause complications, including [anemia](https://my.clevelandclinic.org/health/diseases/3929-anemia) (not enough red blood cells to carry oxygen throughout the body), bone disease, brain damage, [edema](https://my.clevelandclinic.org/health/diseases/12564-edema) (swelling), fluid in and around the lungs, high levels of certain minerals (potassium or phosphorus), infections, nerve damage, [seizures](https://my.clevelandclinic.org/health/articles/6998-seizures-first-aid), [stroke](https://my.clevelandclinic.org/health/diseases/5601-stroke-understanding-stroke). At this stage, kidney transplant or dialysis of the type of hemodialysis or peritoneal dialysis becomes necessary for the survival of the person.

According to the Ministry of Health's Center for Specific Disease Management and Kidney Transplant statistics, about 100,000 people in the country with advanced CKD are being treated with blood and peritoneal dialysis. The pain of dialysis and its financial burden for patients with ESRD should be noted in those on dialysis. According to statistics, the cost of each dialysis session for a patient is about $29 and equivalent to $4176 per year [[13](#_ENREF_13)]. By calculating the population of thousands of dialysis patients in the country, it is possible to predict the heavy burden of this disease on the country's health economy. In Iran, unlike other developed countries, financial support is much less and requires increasing attention from insurance companies and the government. Therefore, an analysis of the economic aspects of ESRD seemed necessary due to its prevalence and high treatment costs in Iran.

**Section 2: Kidney transplant Scenarios**

**Scenario 1:** Assume that you have a chronic kidney disease problem that can be treated with a kidney transplant, while the success rate of the transplant is 10%, so how much are you willing to pay?

**Question 1.** What is the maximum amount you can afford to pay for a kidney transplant?

**Double-bounded dichotomous-choice**

**Question 2.** Do you want to pay 1740 $ for a kidney transplant?

1. Yes (Question 4) 2. No (Question 3)

**Question 3.** Do you want to pay 870 million tomans for a kidney transplant? (Half of the first offer)

1. Yes 2. No

**Question 4.** Do you want to pay 3480 $ for a kidney transplant? (Double the first offer)

1. Yes 2. No

**Scenario 2:** Assume that you have a chronic kidney disease problem that can be treated with a kidney transplant, while the success rate of the transplant is 30%, so how much are you willing to pay?

**Question 1.** What is the maximum amount you can afford to pay for a kidney transplant?

**Double-bounded dichotomous-choice**

**Question 2.** Do you want to pay 1740 $ for a kidney transplant?

1. Yes (Question 4) 2. No (Question 3)

**Question 3.** Do you want to pay 870 million tomans for a kidney transplant? (Half of the first offer)

1. Yes 2. No

**Question 4.** Do you want to pay 3480 $ for a kidney transplant? (Double the first offer)

1. Yes 2. No

**Scenario 3:** Assume that you have a chronic kidney disease problem that can be treated with a kidney transplant, while the success rate of the transplant is 70%, so how much are you willing to pay?

**Question 1.** What is the maximum amount you can afford to pay for a kidney transplant?

**Double-bounded dichotomous-choice**

**Question 2.** Do you want to pay 1740 $ for a kidney transplant?

1. Yes (Question 4) 2. No (Question 3)

**Question 3.** Do you want to pay 870 million tomans for a kidney transplant? (Half of the first offer)

1. Yes 2. No

**Question 4.** Do you want to pay 3480 $ for a kidney transplant? (Double the first offer)

1. Yes 2. No

**Scenario 1:** Assume that you have a chronic kidney disease problem that can be treated with a kidney transplant, while the success rate of the transplant is 99%, so how much are you willing to pay?

**Question 1.** What is the maximum amount you can afford to pay for a kidney transplant?

**Double-bounded dichotomous-choice**

**Question 2.** Do you want to pay 1740 $ for a kidney transplant?

1. Yes (Question 4) 2. No (Question 3)

**Question 3.** Do you want to pay 870 million tomans for a kidney transplant? (Half of the first offer)

1. Yes 2. No

**Question 4.** Do you want to pay 3480 $ for a kidney transplant? (Double the first offer)

1. Yes 2. No

**Section 3: Participants' socioeconomic information**

| Gender | Male |
| --- | --- |
|  | Female |
| employment status | Unemployed |
|  | Employed |
| Health insurance status | No insurance coverage |
|  | Health insurance (rural insurance) |
|  | Health insurance (other than rural insurance) |
|  | Social Security |
|  | Armed Forces |
|  | Other |
| Age | 0-19 years |
|  | 20-44 years |
|  | 45-64 years |
|  | 65-75 years |
| Preference for a kidney preparation | Buy from a healthy person |
|  | Gifts from the deceased |
|  | Gifts from family and friends |
| Awareness of kidney function | I don’t know |
|  | Less than 60% |
|  | Between 30 and 50% |
|  | Between 15 and 30% |
|  | Less than 15% |
| The average monthly income of the household |  |

The mentioned statistics were related to the information of the patients under study in this research. For this reason, it should have been given in the results section.
